# Identification of Potential Predictors of Prognosis and Sorafenib-Associated Survival Benefits in Patients with Hepatocellular Carcinoma after Transcatheter Arterial Chemoembolization

**DOI:** 10.3390/curroncol30010038

**Published:** 2022-12-29

**Authors:** Kun He, Zelong Yang, Xinyu Liu, Yanling Yang, Wenjie Song, Shangyu Wang, Yong Chen

**Affiliations:** Department of Hepatobiliary Surgery, Xijing Hospital, Fourth Military Medical University, Xi’an 710032, China

**Keywords:** predictors, hepatocellular carcinoma, vascular endothelial growth factor, transcatheter arterial chemoembolization, sorafenib

## Abstract

Some studies have shown that sorafenib could significantly prolong the overall survival of patients with unresectable hepatocellular carcinoma treated with transcatheter arterial chemoembolization (TACE). However, other studies revealed that patients had no access to sorafenib-related survival benefits after TACE. To identify the predictive biomarkers of therapeutic efficacy of sorafenib, we explored the potential predictive value of vascular endothelial growth factor (VEGF) and other clinical variables for survival benefits from sorafenib in patients treated with TACE previously. The results demonstrated that patients with tumor size > 7 cm or total bilirubin ≤ 17.3 μmol/L showed significant survival benefits from sorafenib after TACE treatment compared with those with tumor size ≤ 7 cm or total bilirubin > 17.3 μmol/L. Meanwhile, patients with VEGF > 131.09 pg/mL may obtain sorafenib-associated survival benefits after TACE when compared to those with VEGF ≤ 131.09 pg/mL, which needs further confirmation. The abovementioned results are helpful to confirm the specific population who are sensitive to targeted therapy. (1) Background: VEGF plays a crucial role in modulating proliferation and metastasis in HCC. We aimed to explore the relationship between VEGF and the prognosis, as well as the mortality risk of HCC patients who received TACE, and whether it and other variables could be considered as potential biomarkers for predicting the benefits from sorafenib. (2) Method: A total of 230 consecutive newly diagnosed patients with unresectable HCC treated with either TACE or TACE–sorafenib were collected retrospectively. Cox regression analyses were performed to evaluate the prognostic value of VEGF. Furthermore, restricted cubic splines were fitted to assess the nonlinear associations between VEGF and OS, and the threshold effect analysis was subsequently performed. Lastly, the potential factors for predicting the survival benefits from sorafenib after the TACE procedure were identified using the Cox proportional hazard model with an interaction term. (3) Results: VEGF was recognized as an independent prognostic factor for OS in the TACE alone cohort (HR = 3.237, *p* = 0.013). A nonlinear relationship was observed between VEGF and OS in HCC patients with TACE administration after adjustment for confounders (*p* for nonlinearity = 0.030); the mortality risk increased with increasing the baseline VEGF before the inflection point, and the HR for death was 1.008. There was no significant interaction between the VEGF levels and treatment modality (*p* for interaction = 0.233), and further studies are needed to identify its predictive value on the efficacy of sorafenib. Patients with tumor size > 7 cm or total bilirubin ≤ 17.3 μmol/L derived significant sorafenib-related benefits in OS when compared to those with tumor size ≤ 7 cm or total bilirubin > 17.3 μmol/L (*p* for interaction = 0.004 and 0.031, respectively). (4) Conclusions: Within a certain concentration range, elevated baseline VEGF meant an increased risk of death in HCC patients treated with TACE. Significant improvements in OS associated with sorafenib were observed in patients with higher tumor size and lower total bilirubin after TACE treatment.

## 1. Introduction

Hepatocellular carcinoma (HCC) is a highly heterogeneous malignancy, which represents 80–90% of primary liver cancers and ranks third amongst all cancer-related deaths worldwide [1]; despite decades of diagnostic and therapeutic efforts, the long-term prognosis of HCC patients is still dismal with the 5 year survival rate below 20% [2,3]. These disappointing consequences are mainly due to most HCC patients being initially diagnosed at an advanced stage, which prevents them from being candidates for surgery or liver transplantation [4,5].

Transcatheter arterial chemoembolization (TACE) is well recognized as the standard treatment for patients with intermediate-stage HCC [6]. The Barcelona Clinic Liver Cancer (BCLC) stage system advocates that TACE is the main option for HCC patients with BCLC stage B [7,8]. However, due to the high heterogeneity and diversity of tumor burden in patients with intermediate-stage HCC, the median overall survival (OS) of patients treated with TACE monotherapy varies obviously [9,10]. Therefore, combination therapy that involves TACE and other therapies is suggested to derive greater survival benefits. Sorafenib, an oral inhibitor of multiple receptor tyrosine kinases, which include platelet-derived growth factor receptor (PDGFR) and vascular endothelial growth factor receptor (VEGFR) signaling, has been considered as the first-line systemic therapy for advanced HCC patients [11,12]. Several studies have shown that the tumor hypoxia response induced by TACE promotes VEGF expression [13,14,15]; furthermore, high levels of VEGF concentrations indicated poor prognosis in HCC [16]. On the basis of the above facts, researchers envisaged that the antiangiogenic effect of sorafenib may prevent tumor proliferation induced by increased serum VEGF. Unfortunately, the therapeutic superiority of combination therapy over TACE alone remains controversial.

Some studies have demonstrated that TACE–sorafenib obtained significant survival benefits when compared to TACE monotherapy [17,18,19,20,21]. A meta-analysis suggested that the TACE–sorafenib treatment did not prolong OS and only improved time to progression (TTP) [22], the TACTICS trial showed significant improvement in progression-free survival PFS [23], and there was no sorafenib-related survival benefit was observed in the SPACE study [24]. The above evidence is presented in Supplementary Table S1. We summarize that, in addition to differences in population distribution and duration of sorafenib treatment, the more important reason for these inconsistencies is that the need to confirm an available biomarker that can predict the therapeutic effects of sorafenib remains unmet. A previous randomized controlled trial (RCT) of sorafenib versus placebo explored the prognostic value of serum VEGF and its predictive value for the sorafenib benefits in patients with advanced HCC and found that VEGF can independently influence the prognosis of HCC patients but failed to predict the survival benefits from sorafenib [25]. Another RCT study revealed that magnitude improvements in OS in response to sorafenib were observed in patients with hepatitis C virus, with low neutrophil-to-lymphocyte ratio (NLR), or without extrahepatic spread (EHS) [26]. To our knowledge, there is no study that has previously described the role of VEGF in predicting the sorafenib-related survival benefits in unresectable HCC patients after receiving TACE. On the basis of the above discussions, the primary objectives of the retrospective cohort study that we conducted were to (i) reassess the survival difference between combination therapy and TACE alone according to our data, (ii) explore the prognostic and predictive value of VEGF and other clinical characteristics in HCC patients treated with TACE, and (iii) estimate the association between baseline VEGF concentrations and the risk of death in patients with unresectable HCC.

## 2. Materials and Methods

### 2.1. Patients

From October 2018 to March 2021, we retrospectively collected 230 consecutive newly diagnosed patients with unresectable HCC in the Xijing Hospital, who were administrated with either TACE alone or TACE plus sorafenib as the initial treatment of HCC, which was diagnosed on the basis of either histological examination or imaging, according to practice guidelines established by the European Association for the Study of the Liver (EASL) and the American Association for the Study of Liver Diseases (AASLD) [4,6]. In our study, all classifications for patients with HCC were according to EASL Clinical Practice Guidelines. The inclusion criteria of our study were as follows: (i) 18 years or older; (ii) Eastern Cooperative Oncology Group performance status (ECOG PS) ≤ 1; (iii) patients with unresectable HCC and treated with TACE alone or TACE–sorafenib; (iv) Child–Pugh liver function from A5 to B7; (v) at least one measurable lesion with a diameter > 1 cm. The exclusion criteria were as follows: (i) any prior regional or systemic therapies, including resection, ablation, radiation, and targeted molecular therapy; (ii) concomitant with other malignancies; (iii) spontaneous tumor rupture; (iv) contraindications of TACE or sorafenib treatment; (v) partial hepatectomy or liver transplantation after TACE; (vi) tumor size ≤ 5 cm at BCLC stage A. This study was approved by the Institutional Review Board of Xijing Hospital of Fourth Military Medical University (KY20222266-F-1).

### 2.2. Treatment Protocol

Baseline imaging information was collected to evaluate the tumor status before the initial TACE session, and the TACE procedure was performed as follows: a 5F-RH catheter was inserted into the right femoral artery and immediately afterward fed into the hepatic artery selectively to clarify the tumor’s location, size, number, and blood supply through a digital subtraction angiography image. Firstly, 1.0 g of 5-fluorouracil and 50 mg of lobaplatin were injected into the proper hepatic artery for infusion chemotherapy. Subsequently, a microcatheter was super-selectively inserted into the tumor-feeding vessels located in a segment or subsegment. Finally, a DC Bead loaded with 40 mg of epirubicin was injected into the feeder’s vessels for embolization until the blood flow stopped after 5–10 successive cardiac cycles. After the embolization, angiography was analyzed again to confirm complete blood flow occlusion. The type and dose of chemotherapy drugs and lipiodol were determined by tumor burden, blood supply, body surface area, and performance status. All procedures were undertaken by clinicians with more than 10 years of experience. To evaluate the necessity of a consecutive TACE treatment, 4–6 weeks after the initial TACE session, CT or MRI and laboratory examinations (including blood routine, hepatic function, and tumor markers) were performed. The second treatment of TACE was scheduled if follow-up imaging showed intrahepatic residual viable tumors. If the CT or MRI images showed no viable tumor, TACE was discontinued, and the patient was re-evaluated by laboratory tests and imaging (CT or MRI) at 4 week and 8 week intervals, respectively. If new lesions were revealed, subsequent TACE was administered again. 

Patients received sorafenib (Bayer Healthcare, Leverkusen, Germany) twice daily at the initial dose of 400 mg in 4–7 days after the initial TACE. Dose reduction (from 400 mg per day to 400 mg every second day) or treatment interruption due to drug-related adverse events (AEs) was allowed. Continuing the sorafenib therapy was encouraged unless unacceptable drug toxicity or unmanageable disease progression occurred. Sorafenib was discontinued 3 days before each on-demand TACE session to protect liver function.

### 2.3. Outcomes

The primary study endpoint was overall survival (OS), which was defined as the interval of time from the initial HCC diagnosis to death or the last follow-up date. The secondary study endpoint was PFS, which was defined as the interval from the initial diagnosis to imaging progression according to the modified Response Evaluation Criteria in the Solid Tumors (mRECIST) criteria [27], death, or the last follow-up.

### 2.4. Radiographic Evaluation

Measurement of target lesions (*n* ≤ 2) and evaluation of tumor response were performed by each contrast-enhanced CT or MRI by two radiologists (S.W.Z. and L.C.), based on mRECIST criteria; the radiologists have no access to other clinical records of patients, and any inconsistencies between their assessment outcomes were addressed by reaching a mutual consensus. The objective response rate (ORR) was defined as the proportion of patients achieving the best response of partial response (PR) or complete response (CR), and the disease control rate (DCR) was defined as the proportion of patients achieving PR, CR, or stable disease (SD). Sorafenib-related AEs were graded by the National Cancer Institute Common Terminology Criteria for Adverse Events 4.0 [28].

### 2.5. Serological VEGF-A and Other Indicators Measurements

Peripheral venous blood samples (approximately 5 mL) were obtained from HCC patients before the first TACE session and stored at room temperature (37 °C) until use, and then drawn into a serum separator tube and centrifuged at 3500 rpm for 10 min. The supernatant was removed and snap-frozen to −20 °C for storage until analysis. Concentrations of serum VEGF-A were detected using a Vascular Endothelial Growth Factor Assay Kit (chemiluminescence) and JR-1 Chemiluminescent Immunoassay Analyzer (Shandong Weigao Group Medical Polymer Co., Ltd., Weihai, China) according to the instructions of the manufacturer.

For statistical analysis, diagnostics, baseline demographic characteristics, and therapeutic modalities of patients with HCC were collected, including age, ECOG PS, gender, etiologic, Child–Pugh score, ascites, tumor size, number of tumors, portal vein tumor thrombus (PVTT), EHS, BCLC stage, VEGF, alpha-fetoprotein (AFP), total bilirubin, NLR, aspartate aminotransferase (AST), alanine aminotransferase (ALT), and alkaline phosphatase (ALP). There were no missing data for all patients at baseline. All the laboratory and clinical information mentioned above was retrospectively collected from the electronic case system of patients.

### 2.6. Statistical Analysis

There were no censored data for all patients at baseline. Statistical analysis was conducted by R version 4.1.1 (The R Foundation for Statistical Computing) and SPSS version 26.0 (IBM Corp., Armonk, NY, USA), and two-tailed *p*-values less than 0.05 were considered statistically significant. Continuous variables were expressed as the mean ± standard deviation (SD) or median with interquartile range (IQR), and categorical variables were presented as absolute frequencies with percentages. When comparing the baseline data between the two cohorts, the χ^2^ test or Fisher exact test was conducted for categorical variables, and the t-test or the Mann–Whitney U test was performed for continuous variables. The optimal baseline for the VEGF cutoff was selected by using the maximally selected rank statistics [29]. For other continuous laboratory variables, their median was used as a cutoff value: 400 ng/mL for AFP, 17.3 μmol/L for total bilirubin, 2.97 for NLR, and 124 U/L for ALP.

The Kaplan–Meier method and the log-rank test were utilized to compare the survival between the two treatment cohorts. Univariate and multivariate Cox regression analyses were performed to evaluate the prognostic value for VEGF. The association between baseline VEGF concentrations and benefits from sorafenib treatment was assessed on the basis of a stratified Cox regression model with an interaction test; the above analysis procedures were also applied to other clinical variables.

In order to clarify the relationship between baseline VEGF levels (continuous data) and mortality risk in HCC patients, three-knot restricted cubic splines before and after adjustment for confounders were fitted, and the nonlinearity tests were carried out subsequently. A recursive approach was performed to confirm the inflection points associated with the risk of death. Then, the threshold effect of VEGF on OS was evaluated by using smooth scatter and a two-piece-wise Cox regression [30]. Furthermore, one-line linear regressions were compared based on log-likelihood ratio tests. Lastly, logistic regression was conducted to explore the differences in survival at different time points in HCC patients with high and low serum VEGF concentrations.

## 3. Results

### 3.1. Baseline Characteristics and Follow-Up of Unresectable HCC Patients

**Figure 1:**
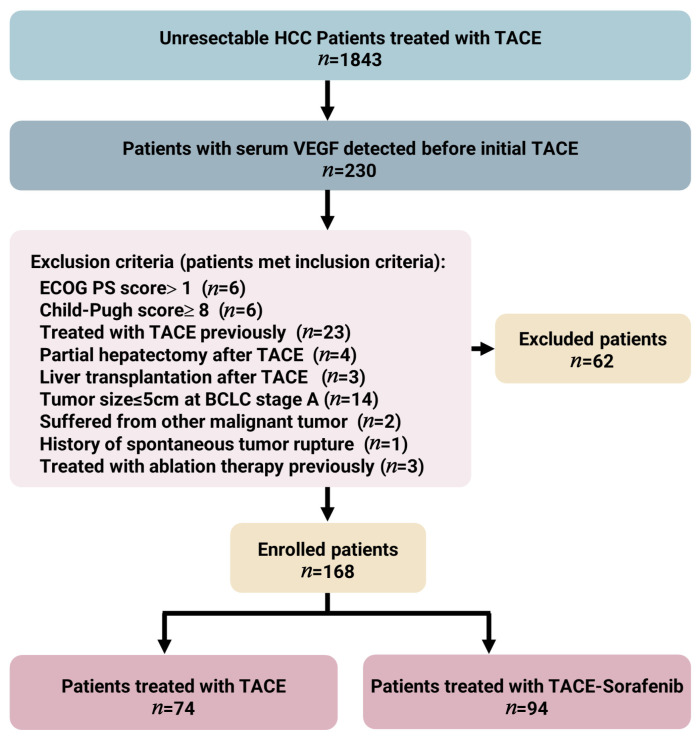
The flowchart of the study; “*n*” represents the number of patients.

In total, 168 patients fulfilled the inclusion/exclusion criteria, 74 of whom underwent TACE and 94 of whom underwent TACE–sorafenib as their initial treatment (Figure 1). The baseline demographics and clinical characteristics between the TACE cohort and the TACE–sorafenib cohort were comparable (Table 1). The etiological cause of HCC patients in both cohorts was chronic viral hepatitis B (82.4% and 74.5%, respectively). The median follow-up time was 18.6 (1.3–35.4) months for the TACE cohort and 21.4 (3.0–35.1) months for the TACE–sorafenib cohort. The median time between first TACE and initial sorafenib treatment in the TACE–sorafenib cohort was 4.8 (3.6–6.2) days, and the median time of sorafenib therapy was 7.0 (4.0–15.0) months. A total of 39 (52.7%) patients received repeated TACE at a mean of 1.3 (1–3) times in the TACE cohort, and 54 (57.4%) patients received repeated TACE at a mean of 1.6 (1–4) times in the TACE–sorafenib cohort.

### 3.2. Survival Analysis and Tumor Response

**Figure 2:**
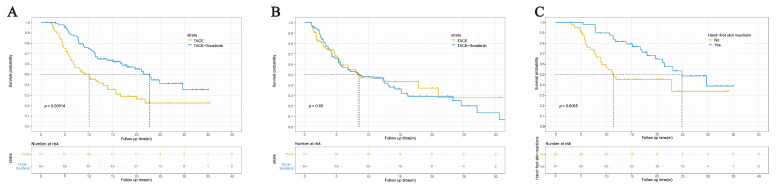
Kaplan–Meier survival curves of unresectable HCC patients. (**A**) Kaplan–Meier overall survival (OS) curves for two cohorts. (**B**) Kaplan–Meier progression-free survival (PFS) curves for two cohorts. (**C**) Kaplan–Meier OS curves for patients with or without hand-foot skin reactions in the TACE–sorafenib cohort.

The median OS was 22.8 (18.8–26.9) months in the TACE–sorafenib cohort and 10.1 (6.5–13.7) months in the TACE cohort (HR = 0.454, 95% CI = 0.299–0.688, *p* < 0.001) (Figure 2A). However, for PFS, no statistical difference between the two cohorts was observed (HR = 1.089, 95% CI = 0.711–1.668, *p* = 0.695) (Figure 2B). The DCR was higher in the TACE–sorafenib cohort (93.6%) than in the TACE cohort (85.1%), for ORR; the result was similar (70.2% vs. 55.4%). The detailed information is summarized in Supplementary Table S2.

### 3.3. Adverse Events Attributed to Sorafenib

In Supplementary Table S3, the sorafenib-related adverse events (AEs) in patients treated with combination therapy were revealed. Among 94 HCC patients, 72 (76.6%) patients suffered 104 AEs. The most common AE was hand/foot skin reactions (54.3%), followed by diarrhea (23.4%) and rashes (5.3%). Dose reductions or temporary interruptions of sorafenib occurred in 48 (51.1%) patients. A total of 18 (19.1%) patients discontinued sorafenib due to intolerance, drug-related toxic effects, disease progression, and hepatic decompensation. No drug-related deaths were recorded during the entire period of sorafenib treatment. Patients in the TACE–sorafenib cohort with hand–foot skin reactions had significantly longer survival than those without; their median OS was 24.9 (17.1–32.8) months and 11.4 (3.0–19.8) months, respectively (HR = 0.443, 95% CI = 0.243–0.809, *p* = 0.006) (Figure 2C).

### 3.4. Prognostic Value of Serum VEGF

**Figure 3:**
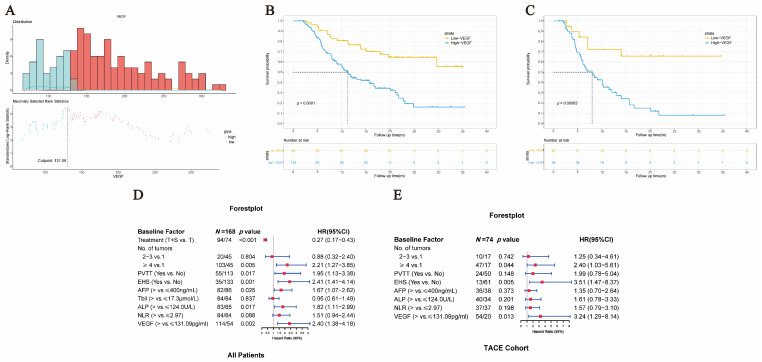
The optimal cutoff value for VEGF and prognosis factors for unresectable HCC. (**A**) The optimal cutoff value for VEGF based on the maximally selected rank statistics. Kaplan–Meier overall survival (OS) curves for baseline VEGF (**B**) in all patients and (**C**) in the TACE cohort. Prognosis factors for unresectable HCC (**D**) in all patients and (**E**) in the TACE cohort based on multivariate Cox regression analysis.

The maximally selected rank statistics analysis showed that the optimal cutoff point for VEGF was 131.09 pg/mL (Figure 3A). The univariate Cox analysis demonstrated that high baseline serum VEGF (>131.09 pg/mL), treatment modality, number of tumors (≥4), presence of PVTT, EHS, high AFP (>400 ng/mL), elevated total bilirubin (>17.3 μmol/L), high NLR (>2.97), and high ALP (>124 U/L) were correlated with worse OS significantly in all patients (Table 2); the median OS of all patients with high and low baseline VEGF was 11.3 months and not reached (NR) (HR = 2.994, 95% CI = 1.773–5.055, *p* < 0.001) (Figure 3B). In the TACE cohort with high and low baseline VEGF, the median OS was 7.97 months and NR (HR = 4.053, 95% CI = 1.702–9.648, *p* < 0.001) (Figure 3C), and VEGF, number of tumors (≥4), presence of PVTT, EHS, high AFP, high NLR, and high ALP were associated with worse OS in this cohort.

The multivariate Cox analysis showed that high baseline serum VEGF, the number of tumors (≥4), the presence of PVTT, EHS, elevated AFP, and high ALP could independently impact poor prognosis in all patients. In addition, high baseline serum VEGF, the number of tumors (≥4), and presence of EHS were identified as independent prognostic factors of poor survival in the TACE cohort (Figure 3D,E).

### 3.5. Nonlinear Association and Threshold Effect of Baseline VEGF on OS

**Figure 4:**
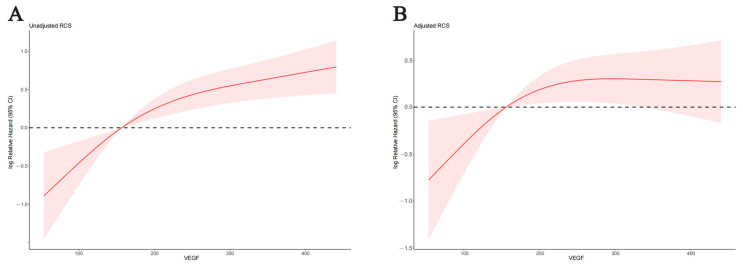
Three-knot restricted cubic splines of the relationship between the baseline VEGF and risk of death. The restricted cubic spline of VEGF (**A**) before and (**B**) after adjustment for confounders.

The relationship between baseline VEGF and mortality risk of unresectable HCC before adjustment for potential confounders is shown in Figure 4A, and a significant threshold effect of VEGF on OS could be observed (*p* for nonlinearity= 0.032). The HR before and after the turning point (189.79 pg/mL) was 1.009 (95% CI = 1.004–1.015, *p* = 0.001) and 1.002 (95% CI = 1.000–1.003, *p* = 0.013), respectively. In Figure 4B, the relationship between baseline VEGF and mortality risk of HCC after adjustment for confounders including treatment modality, number of tumors, PVTT, EHS, AFP, total bilirubin, ALP, and NLR was revealed; the threshold effect of VEGF on OS was still obvious (*p* for nonlinearity = 0.030), the HR before and after the turning point (189.79 pg/mL) was 1.008 (95% CI = 1.002–1.014, *p* = 0.009) and 1.000 (95% CI = 0.998–1.002, *p* = 0.862), respectively. Detailed results of threshold effect analysis are presented in Table 3. According to the above information, we could learn that, whether or not the effects of confounders were adjusted, the risk of death in patients with unresectable HCC increased with the increasing value of baseline VEGF before the turning point. On the other hand, after the turning point, with the VEGF concentration increasing, the mortality risk also increased when confounders were unadjusted but it plateaued after confounders were adjusted.

### 3.6. Predictive Value of Serum VEGF and Other Clinical Characteristics

**Figure 5:**
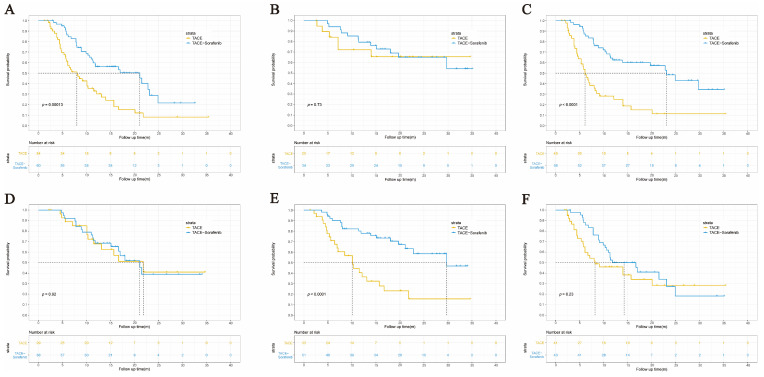
Potential factors for predicting the benefits from sorafenib after TACE administration. (**A**) High VEGF and (**B**) low VEGF (*p* for interaction = 0.233). (**C**) Tumor size > 7 cm and (**D**) tumor size ≤ 7 cm (*p* for interaction = 0.004). (**E**) Low total bilirubin and (**F**) high total bilirubin (*p* for interaction = 0.031).

The interaction analysis between the therapeutic modality and each subgroup was performed to identify whether baseline VEGF and other clinical variables could predict the sorafenib benefits (Table 4). Although there was no significant interaction between the VEGF levels and treatment modality (*p* for interaction = 0.233), we could learn from Figure 5A,B that the OS of patients with high levels of VEGF was significantly prolonged after receiving TACE–sorafenib, while patients with low levels of VEGF had no prolonged OS. A larger sample size is needed to further verify the predictive value of VEGF in response to sorafenib treatment. Interestingly, we were surprised to learn that patients with a tumor size > 7 cm showed significant benefits from sorafenib in OS when compared to those with a tumor size ≤ 7 cm (*p* for interaction = 0.004) (Figure 5C,D). Conversely, HCC patients with total bilirubin ≤ 17.3 μmol/L derived greater sorafenib-related survival benefits than those with total bilirubin > 17.3 μmol/L (*p* for interaction = 0.031) (Figure 5E,F). Then, we applied this VEGF cutoff value to the patients with BCLC stage B and C in two cohorts. A trend toward benefits related to sorafenib was also observed in patients with VEGF > 131.09 pg/mL (*p* for interaction = 0.305) (Supplementary Figure S1A,B). 

### 3.7. Comparison of Mortality Based on Different VEGF Levels

To further explore the relationship between different baseline VEGF levels and survival in patients with unresectable HCC, we assessed the differences in mortality between patients with high and low VEGF (>131.09 vs. ≤131.09 pg/mL) levels at 6 months, 1 year, and 2 years using univariate and multivariate logistic regression; the results demonstrated that patients with high VEGF had a higher risk of death at 1 year and 2 years than those with low VEGF (Supplementary Table S4).

## 4. Discussion

After retrospective analysis, we found that patients in the TACE–sorafenib cohort had significantly improved OS than those who received TACE alone (*p* < 0.05), which was consistent with these prior studies [17,18,19,20,21]. However, no insignificant difference was observed between the two cohorts for PFS in our study, which was in agreement with the result of a phase III RCT [31]. Masatoshi Kudo et al. advocated that combining TACE with sorafenib could lead to better antitumor efficacy and significantly prolonged PFS compared to TACE monotherapy. However, it could not be considered that our observation conflicted with the TACTICS trial as this RCT did not compare OS due to insufficient OS-reached events. Furthermore, the TACTICS trial had a protocol of treating patients with an initial round of sorafenib followed by TACE. In contrast, our treatments were in a reversed order such that this inconsistency may be feasible. This indicates a potentially more effective treatment sequence, which should be explored further. There were also several RCT studies performed to combine TACE with molecule-targeted drugs such as sorafenib, brivanib, and orantinib, which failed to prove any survival benefits [24,31,32,33,34]. Therefore, further investigations are required to confirm whether the combination therapy of TACE–sorafenib could provide greater improvements in OS than TACE monotherapy for patients with advanced HCC. The calculated DCR and ORR were similar to the findings of the SPACE trial and two retrospective studies [20,21,24]. Notably, the high DCR and ORR in both groups were probably due to the super-selective nature of the TACE procedure.

Hand/foot skin reactions were among the most frequent AEs of sorafenib [35]; we showed a greater magnitude of survival benefits in patients with skin reactions when compared to those without. This result was also confirmed by a previous study [36]. An established clinical prognostic model including dermatologic AEs performed well in the internal validation with Harrel’s c index of 0.73 [37]. A presumable cause for this survival difference could lie in the interference of VEGFR signaling pathway due to sorafenib [38], which leads to the inhibition of tumor metastasis and proliferation.

VEGF is the most common and critical angiogenic regulator under pathological or physiological conditions [39], and it also has major repercussions for the prognosis of HCC patients. Serum VEGF levels could not only independently affect the prognosis of patients with HCC but were also closely associated with its proliferation and metastasis [40,41,42]. In addition, the early decreased VEGF concentrations were also related to better OS and PFS [43]. As previously described, the circulating VEGF levels increased after the TACE procedure [14]; we found that high baseline VEGF (>131.09 pg/mL) was an independent prognostic factor for poor survival in HCC patients treated with TACE, which corroborated the above views. We also revealed that the 1 year and 2 year survival rates of patients with high VEGF were significantly lower than those with low VEGF. 

A threshold effect analysis was conducted to further investigate the relationship between VEGF and the risk of death in HCC patients who underwent TACE; we demonstrated that, before the inflection point (189.79 pg/mL), a 1 pg/mL increase in baseline VEGF was associated with 0.8% mortality risk in HCC patients previously treated with TACE after confounding factors were adjusted. In contrast, after the inflection point, the death risk of HCC patients remained mostly stable, which suggested that, although high VEGF levels were closely correlated with poor prognosis of HCC, it was not that a higher concentration of VEGF led to a shorter survival of patients.

We analyzed the interactions between baseline VEGF and other clinical variables, as well as treatment modalities (TACE alone or TACE–sorafenib). We only demonstrated the trend of survival benefits related to sorafenib in the subgroup of patients with high VEGF (*p* for interaction = 0.233), which was similar to the results of a previous study (sorafenib versus placebo) [25], but we did observe the prolongation of survival of patients with high VEGF after sorafenib administration; furthermore, in patients with low VEGF levels, the survival benefits brought by sorafenib were not shown. This finding is consistent with a previous study showing that sorafenib is more sensitive to HCC cells with VEGF gene amplification [44], which may be due to VEGF modulating the efficacy of sorafenib treatment in the forms of autocrine or paracrine secretion [45,46]. Thus, a larger sample size is needed to further explore the predictive value of VEGF for benefits from sorafenib in HCC patients previously treated with TACE. 

Conversely, it was gratifying to note that patients with tumor size > 7 cm or total bilirubin ≤ 17.3 μmol/L achieved significant sorafenib-related survival benefits (*p* for interaction = 0.004 and 0.031, respectively). A previous study also showed that the survival of advanced HCC patients with the largest tumor < 7 cm who continued to receive sorafenib after TACE could not be further improved [32]. A multicenter retrospective study focused on screening candidates for TACE–sorafenib combination therapy, which indicated that patients with relatively high tumor burden derived greater benefits from sorafenib [47]. The radiological response rates decrease with the increase in tumor burden [48]. On the basis of the studies presented above, we speculated that patients with tumors with relatively small diameters may not need to receive sorafenib sequentially after the TACE procedure.

According to a subset analysis of the Asia–Pacific trial [49], sorafenib-related survival benefits were observed in HCC patients with baseline bilirubin levels less than three times upper limit of normal (<3.0 mg/dL). However, the liver function of patients with advanced HCC was highly heterogeneous [50]; deterioration of liver function shortened the treatment duration of sorafenib and, accordingly, the survival benefits may have been decreased [51]. In addition, bilirubin inhibited sorafenib’s anticancer activity by blocking MCL-1 degradation in HCC cells [52]. To sum up, the benefits from sorafenib would be lower in patients with poor liver function when compared to those with good liver function [53]. In our study, the median OS of patients with low total bilirubin was significantly longer than those with high total bilirubin.

The study that we conducted had some limitations that must be noted. Firstly, this was a retrospective single-center cohort study; hence, the retrospective confounding bias is inevitable, and more research is needed to confirm our findings. Secondly, the majority of HCCs in our study were HBV-related. However, in other countries, HCV-related or NAFLD-related HCC is more common [54]. Thirdly, due to the limitation induced by the sample size, we were unable to verify HCV, which was a potential predictor of the sorafenib-associated benefits reported in a previous study [26]. 

## 5. Conclusions

We retrospectively analyzed the survival difference of 168 unresectable HCC patients treated with TACE alone or TACE–sorafenib and found that the OS of patients receiving combination treatment was significantly prolonged. In the TACE–sorafenib cohort, patients with hand/foot skin reactions related to sorafenib had a significant survival benefit compared with those without. After confirming that VEGF was an independent prognostic factor for patients receiving TACE, we also revealed that, when baseline serum VEGF < 189.79 pg/mL, the risk of death increased in HCC patients with increasing concentrations of VEGF. Lastly and foremost, our results demonstrated that in patients with TACE administration, larger tumor size and lower total bilirubin were predictors of sorafenib-associated survival benefits, which provides a reference value for identifying sensitive populations of unresectable HCC patients receiving targeted therapy. The predictive value of serum VEGF for the efficacy of sorafenib after TACE treatment needs further confirmation.

## Figures and Tables

**Table 1:** Baseline demographics and clinical characteristics of patients in two cohorts.

Characteristic	TACE Cohort (*n* = 74)	TACE–Sorafenib Cohort (*n* = 94)	*p*-Value
Gender			0.167
Male	63 (85.1)	72 (76.6)	
Female	11 (14.9)	22 (23.4)	
Age (years)	54.15 ± 12.03	53.68 ± 10.79	0.791
ECOG PS			0.996
0	63 (85.1)	80 (85.1)	
1	11 (14.9)	14 (14.9)	
Etiologic cause			0.406
Hepatitis B	61 (82.4)	70 (74.5)	
Hepatitis C	1 (1.4)	4 (4.2)	
Other	12 (16.2)	20 (21.3)	
Child–Pugh score			0.060
5	53 (71.6)	81 (86.2)	
6	18 (24.3)	11 (11.7)	
7	3 (4.1)	2 (2.1)	
BCLC stage			0.752
A	8 (10.8)	13 (13.8)	
B	36 (48.6)	41 (43.6)	
C	30 (40.6)	40 (42.6)	
Tumor size (cm)			0.745
≤5 cm	15 (20.3)	21 (22.3)	
>5 cm	59 (79.7)	73 (77.7)	
No. of tumors			0.574
1	17 (23.0)	28 (26.8)	
2–3	10 (13.5)	10 (11.9)	
≥4	47 (63.5)	56 (61.3)	
PVTT			0.940
No	50 (67.6)	63 (67.0)	
Yes	24 (32.4)	31 (33.0)	
EHS			0.355
No	61 (82.4)	72 (76.6)	
Yes	13 (17.6)	22 (23.4)	
Ascites			0.590
No	65 (87.8)	85 (90.4)	
Yes	9 (12.2)	9 (9.6)	
AFP (ng/mL)			0.970
≤400	38 (51.4)	48 (51.1)	
>400	36 (48.6)	46 (48.9)	
Tbil (μmol/L)	17.7 (13.4–25.4)	16.5 (13.5–22.2)	0.307
ALP (U/L)	140 (89–202)	119 (93–154)	0.223
AST (U/L)	51 (32–83)	51 (32–74)	0.506
ALT (U/L)	44 (26–72)	41 (28–62)	0.445
NLR	2.97 (2.03–4.09)	2.98 (2.01–4.86)	0.390
VEGF (pg/mL)	163.58 (119.68–239.82)	154.62 (103.36–226.74)	0.388

ECOG PS, Eastern Cooperative Oncology Group performance status; PVTT, portal vein tumor thrombus; EHS, extrahepatic spread; AFP, alpha-fetoprotein; Tbil, Total bilirubin ALP, alkaline phosphatase; AST, aspartate aminotransferase; ALT, alanine aminotransferase; NLR, neutrophil-to-lymphocyte ratio; VEGF, vascular endothelial growth factor.

**Table 2:** Identification of risk factors for OS based on univariate analysis of the all-patient and TACE cohorts.

	All Patients (*n* = 168)		TACE Cohort (*n* = 74)	
Baseline Factor	HR (95% CI)	*p* Value	HR (95% CI)	*p* Value
Treatment (T + S vs. T)	0.45 (0.30–0.69)	<0.001		
Age (>60 vs. ≤60)	0.91 (0.58–1.43)	0.686	0.97 (0.53–1.79)	0.931
Child–Pugh stage (B vs. A)	1.74 (0.64–4.75)	0.281	0.98 (0.24–4.07)	0.977
Tumor size (>5 vs. ≤5 cm)	1.36 (0.81–2.29)	0.244	1.98 (0.92–4.36)	0.081
No. of tumors				
2–3 vs. 1	0.78 (0.33–1.85)	0.571	1.29 (0.45–3.72)	0.638
≥4 vs. 1	2.12 (1.26–3.55)	0.004	2.68 (1.20–5.98)	0.016
PVTT (yes vs. no)	3.14 (2.05–4.81)	<0.001	4.44 (2.27–8.71)	<0.001
EHS (yes vs. no)	3.79 (2.43–5.91)	<0.001	6.19 (2.97–12.89)	<0.001
AFP (>400 vs. ≤400 ng/mL)	2.06 (1.35–3.13)	0.001	2.05 (1.13–3.71)	0.018
Tbil (>17.3 vs. ≤17.3 μmol/L)	1.55 (1.02–2.35)	0.041	0.92 (0.52–1.64)	0.778
ALP (>124 vs. ≤124.0 U/L)	2.04 (1.34–3.10)	0.001	1.87 (1.04–3.38)	0.038
NLR (>2.97 vs. ≤2.97)	1.93 (1.27–2.94)	0.002	2.44 (1.33–4.48)	0.004
VEGF (>131.09 vs. ≤131.09 pg/mL)	2.99 (1.77–5.06)	<0.001	4.05 (1.70–9.65)	<0.001

T + S, TACE plus sorafenib; T, TACE; PVTT, portal vein tumor thrombus; EHS, extrahepatic spread; AFP, alpha-fetoprotein; Tbil, total bilirubin; ALP, alkaline phosphatase; NLR, neutrophil-to-lymphocyte ratio; VEGF, vascular endothelial growth factor.

**Table 3:** Threshold effect analysis for the baseline serum VEGF based on a two-piece-wise Cox regression model.

Cox Regression Model	Unadjusted HR (95% CI)	Adjusted HR (95% CI)
The one-line Cox regression model	1.003 (1.002–1.004)	1.001 (1.000–1.002)
The two-piece-wise Cox regression model		
≤189.79 pg/mL	1.009 (1.004–1.015)	1.008 (1.002–1.014)
>189.79 pg/mL	1.002 (1.000–1.003)	1.000 (0.998–1.002)
*p* for log-likelihood ratio test	0.016	0.014

Adjusted confounders were as follows: treatment modality, number of tumors, portal vein tumor thrombus (PVTT), extrahepatic spread (EHS), alpha-fetoprotein (AFP), total bilirubin, alkaline phosphatase (ALP), and neutrophil-to-lymphocyte ratio (NLR).

**Table 4:** VEGF and other clinical variables for predicting sorafenib-related survival benefit.

Baseline Factor	*n* (T + S/T)	HR (95% CI)	*p* for Inter-Action
Age			0.587
≤60	119 (68/51)	0.487 (0.296–0.801)	
>60	49 (26/23)	0.397 (0.183–0.858)	
Hepatitis B *			0.663
No	32 (20/12)	0.312 (0.102–0.953)	
Yes	131 (70/61)	0.502 (0.319–0.790)	
BCLC stage			0.620
A ^#^	21 (13/8)	—	
B	77 (41/36)	0.303 (0.154–0.598)	
C	70 (40/30)	0.259 (0.141–0.474)	
Tumor size			0.004
≤7 cm	67 (38/29)	0.965 (0.464–2.006)	
>7 cm	101 (56/45)	0.287 (0.170–0.484)	
No. of tumors			0.281
1	45 (28/17)	0.680 (0.273–1.693)	
2–3	20(10/10)	0.136 (0.016–1.142)	
≥4	103(56/47)	0.361 (0.214–0.608)	
PVTT			0.208
No	113 (63/50)	0.443 (0.253–0.775)	
Yes	55 (31/24)	0.260 (0.130–0.522)	
EHS			0.150
No	133 (72/61)	0.404 (0.241–0.678)	
Yes	35 (22/13)	0.200 (0.086–0.466)	
AFP (ng/mL)			0.835
≤400	86 (48/38)	0.392 (0.207–0.743)	
>400	82 (46/36)	0.458 (0.261–0.801)	
Total bilirubin (μmol/L)			0.031
≤17.3	84 (51/33)	0.279 (0.147–0.527)	
>17.3	84 (43/41)	0.716 (0.410–1.248)	
ALP (U/L)			0.927
≤124	85 (51/34)	0.425 (0.224–0.808)	
>124	83 (43/40)	0.475 (0.273–0.826)	
NLR			0.358
≤2.97	84 (47/37)	0.498 (0.263–0.945)	
>2.97	84 (47/37)	0.326 (0.183–0.580)	
VEGF (pg/mL)			0.233
≤131.09	54 (34/20)	0.833 (0.311–2.232)	
>131.09	114 (60/54)	0.411 (0.257–0.657)	

T + S, TACE plus sorafenib; PVTT, portal vein tumor thrombus; EHS, extrahepatic spread; AFP, alpha-fetoprotein; Tbil, total bilirubin; ALP, alkaline phosphatase; NLR, neutrophil-lymphocyte ratio; VEGF, vascular endothelial growth factor. * Patients with hepatitis C (*n* = 5) were excluded. ^#^ HR (95% CI) was not calculated for patients with BCLC stage A due to small number of patients.

## Data Availability

The original contributions presented in the study are included in the article/Supplementary Materials. Further inquiries can be directed to the corresponding authors.

## Supplementary Materials

The following supporting information can be downloaded at https://www.mdpi.com/article/10.3390/curroncol30010038/s1: Figure S1. Baseline VEGF for predicting the benefits from sorafenib after TACE administration in patients with BCLC stage B and C. (A) High VEGF and (B) low VEGF (*p* for interaction = 0.305); Table S1. Comparison of survival outcomes between TACE–sorafenib and TACE alone for unresectable HCC in previous studies; Table S2. The median OS, PFS, DCR, and ORR in the TACE cohort and the TACE–sorafenib cohort; Table S3. Sorafenib-related adverse events during targeted therapy; Table S4. Unadjusted and adjusted odds ratios (ORs) of mortality with 95% CIs in unresectable HCC patients treated with TACE (> vs. ≤131.09 pg/mL).
