# Supplementary material for: Identification of Potential Predictors of Prognosis and Sorafenib-Associated Survival Benefits in Patients with Hepatocellular Carcinoma after Transcatheter Arterial Chemoembolization
Source: Curr Oncol. 2022 Dec 29;30(1):476–91. doi: 10.3390/curroncol30010038 (PMC9857819; doi:10.3390/curroncol30010038)
Supplement: Supplementary file 1 [file curroncol-30-00038-s001.zip › Figure S1.pdf]

Figure legend

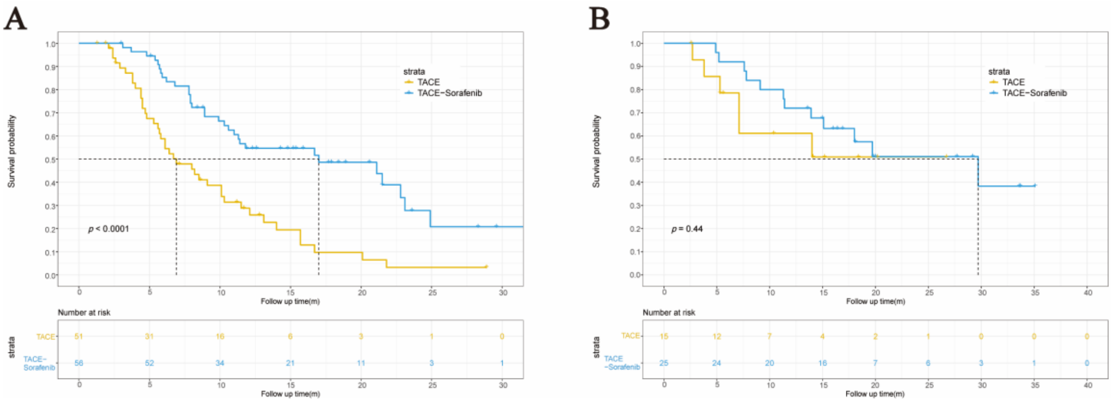

Figure S1: Baseline VEGF for predicting the benefits from sorafenib after TACE administration in patients with BCLC stage B and C. (A) High VEGF and (B) low VEGF (p for interaction=0.305).
