# Supplementary material for: Identification of Potential Predictors of Prognosis and Sorafenib-Associated Survival Benefits in Patients with Hepatocellular Carcinoma after Transcatheter Arterial Chemoembolization
Source: Curr Oncol. 2022 Dec 29;30(1):476–91. doi: 10.3390/curroncol30010038 (PMC9857819; doi:10.3390/curroncol30010038)
Supplement: Supplementary file 1 [file curroncol-30-00038-s001.zip › Table S1.pdf]

Table S1. Comparison of survival outcomes between TACE-Sorafenib and TACE alone for unresectable HCC in previous studies

Studies	OS	PFS	TTP	Ref.
Zhu K et al., 2014.	Yes		Yes	[17]
Li J et al., 2015.	Yes		Yes	[18]
Geschwind JF et al., 2016.	Yes			[19]
Zhang X et al., 2017.	Yes		Yes	[20]
Cai R et al., 2017.	Yes			[21]
Li L et al., 2018.	No		Yes	[22]
Kudo M et al., 2020.	Uncertain	Yes		[23]
Lencioni R et al., 2016.	No		No	[24]

OS, overall survival; PFS, progression-free survival; TTP, time to progression.
