# Supplementary material for: Identification of Potential Predictors of Prognosis and Sorafenib-Associated Survival Benefits in Patients with Hepatocellular Carcinoma after Transcatheter Arterial Chemoembolization
Source: Curr Oncol. 2022 Dec 29;30(1):476–91. doi: 10.3390/curroncol30010038 (PMC9857819; doi:10.3390/curroncol30010038)
Supplement: Supplementary file 1 [file curroncol-30-00038-s001.zip › Table S2.pdf]

Table S2. The median OS, PFS, DCR, ORR in the TACE cohort and the TACE-Sorafenib cohort

	**TACE cohort**	**T-S cohort**	**HR* (95%CI)**	***p* value**
Median OS (m)	10.1 (6.5-13.7)	22.8 (18.8-26.9)	0.454 (0.299-0.688)	<0.001
Median PFS (m)	8.4 (3.0-13.8)	8.6 (2.9-14.2)	1.089 (0.711-1.668)	0.695
DCR (%)	85.1	93.6	-	-
ORR (%)	55.4	70.2	-	-

T-S, TACE-Sorafenib; OS, overall survival; PFS, progression-free survival; DCR, disease control rate; ORR, objective response rate; HR*, TACE-Sorafenib cohort vs. TACE cohort.
