# Supplementary material for: Identification of Potential Predictors of Prognosis and Sorafenib-Associated Survival Benefits in Patients with Hepatocellular Carcinoma after Transcatheter Arterial Chemoembolization
Source: Curr Oncol. 2022 Dec 29;30(1):476–91. doi: 10.3390/curroncol30010038 (PMC9857819; doi:10.3390/curroncol30010038)
Supplement: Supplementary file 1 [file curroncol-30-00038-s001.zip › Table S3.pdf]

Table S3. Sorafenib-related adverse events during targeted therapy (n=94)

Adverse Event	All Grades	Grade 1-2 events	Grade 3-4 events
Hand-foot-skin reactions	51 (54.3)	41 (43.6)	10 (10.6)
Diarrhea	22 (23.4)	15 (16.0)	7 (7.4)
Rash	5 (5.3)	4 (4.3)	1 (1.1)
Hypertension	4 (4.3)	2 (2.1)	2 (2.1)
Arthralgia	4 (4.3)	4 (4.3)	0 (0)
Gingival bleeding	3 (3.2)	3 (3.2)	0 (0)
Fatigue	3 (3.2)	2 (2.1)	1 (1.1)
Anorexia	2 (2.1)	1 (1.1)	1 (1.1)
Alopecia	2 (2.1)	1 (1.1)	1 (1.1)
Epistaxis	1 (1.1)	1 (1.1)	0 (0)
Voice change	1 (1.1)	1 (1.1)	0 (0)
