# Supplementary material for: Identification of Potential Predictors of Prognosis and Sorafenib-Associated Survival Benefits in Patients with Hepatocellular Carcinoma after Transcatheter Arterial Chemoembolization
Source: Curr Oncol. 2022 Dec 29;30(1):476–91. doi: 10.3390/curroncol30010038 (PMC9857819; doi:10.3390/curroncol30010038)
Supplement: Supplementary file 1 [file curroncol-30-00038-s001.zip › Table S4.pdf]

Table S4. Unadjusted and adjusted odds ratio (OR) of mortality with 95% CIs in unresectable HCC patients treated with TACE (> vs. ≤131.09pg/ml)

	Unadjusted OR (95% CI)	Adjusted OR (95% CI)
Mortality at 6-month	3.04 (1.10-8.40)	3.39 (0.99-11.53)
Mortality at 1-year	3.50 (1.67-7.33)	4.03 (1.53-10.64)
Mortality at 2-year	3.59 (1.81-7.15)	3.56 (1.54-8.24)

Adjusted confounders that included as follows: treatment modality, number of tumors, portal vein tumor thrombus (PVTT), extrahepatic spread (EHS), alpha-fetoprotein (AFP), total bilirubin, alkaline phosphatase (ALP), neutrophil-lymphocyte ratio (NLR).
